# Editorial: Proteostasis disruption in neurodegenerative disorders: mechanisms and treatment strategies

**DOI:** 10.3389/fnmol.2026.1796704

**Published:** 2026-02-11

**Authors:** Jonasz Jeremiasz Weber, Hiroaki Taniguchi, Maxim Sokolov

**Affiliations:** 1 Department of Human Genetics, Ruhr University Bochum, Bochum, Germany; 2 Institute of Medical Genetics and Applied Genomics, Eberhard Karls University Tübingen, Tübingen, Germany; 3 Department of Experimental Embryology, Institute of Genetics and Animal Biotechnology of the Polish Academy of Sciences, Jastrzębiec, Poland; 4 African Genome Center, University Mohammed VI Polytechnic (UM6P), Ben Guerir, Morocco; 5 Departments of Ophthalmology, Biochemistry and Molecular Medicine, Neuroscience, West Virginia University, Morgantown, WV, United States

**Keywords:** autophagy, mitochondrial proteostasis, molecular chaperones and protein-unfolding ATPases, neurodegeneration, proteases, proteasome, protein aggregation, transcription of proteasomal genes

Proteostasis—the dynamic balance of protein synthesis, folding, trafficking, and degradation—is essential for cellular homeostasis and organismal health. In neurons, which are long-lived and largely irreplaceable, the maintenance of a functional proteome is particularly critical and vulnerable to age-related decline. Indeed, aging represents the major risk factor for most neurodegenerative disorders, which are characterized by a progressive breakdown of neuronal proteostasis and the accumulation of misfolded and aggregation-prone proteins (Hou et al., 2019). These aberrant protein species can evade or overwhelm cellular quality control systems, disrupt synaptic and mitochondrial function, and trigger neuroinflammatory responses, ultimately leading to neuronal dysfunction and degeneration. In this Research Topic, we bring together six contributions that address key molecular mechanisms underlying neuronal proteostasis failure and explore emerging concepts and therapeutic strategies aimed at restoring protein homeostasis in neurodegenerative disease.

A central feature of proteostasis disruption is the misfolding, self-association/oligomerization, and aggregation of disease-linked proteins, processes that can overwhelm compensatory chaperone-based and proteolytic machineries (Soto, 2003; Soto and Pritzkow, 2018). In recent years, additional layers of complexity have emerged, including the role of liquid–liquid phase separation (LLPS), whereby proteins form liquid-like condensates that may precede aggregation (Babinchak and Surewicz, 2020), as well as the existence of distinct conformational strains associated with differential toxicity. These concepts are comprehensively reviewed by Ruiz-Ortega et al., who focus on α-synuclein aggregation in the context of Parkinson's disease. The authors highlight how diverse cellular environments modulate α-synuclein conformational states and conclude that distinct resulting strains give rise to heterogeneous pathological consequences relevant to the spectrum of synucleinopathies.

Protein misfolding and conformational dysregulation do not necessarily represent irreversible determinants of protein fate, as rescue mechanisms—most prominently molecular chaperones—can assist denatured proteins in refolding toward their native states and thereby protect neurons from proteotoxic stress (Balchin et al., 2016; Lindberg et al., 2015; Muchowski and Wacker, 2005). In their review article, Varte and Rincon-Limas focus on the chaperonin TCP-1 ring complex (TRiC), a folding machinery responsible for the correct folding of approximately 10% of the proteome (Lopez et al., 2015). The authors highlight the role of TRiC in neurological disorders and describe how this complex can modulate the aggregation of various disease-linked proteins. Importantly, they discuss emerging therapeutic strategies that aim to exploit TRiC-dependent mechanisms of protein refolding and disaggregation to counteract neurodegenerative processes.

While proper folding and refolding are critical for maintaining protein integrity, the efficient removal of dysfunctional or surplus proteins is equally essential for proteostasis, both to prevent proteotoxicity and to recycle cellular resources. Neurons rely heavily on ubiquitin-dependent proteasomal degradation and autophagy, processes that are regulated at multiple levels, ranging from transcriptional control to post-translational modifications (Ciechanover and Brundin, 2003; Ciechanover and Livneh, 2025; Le Guerroué and Youle, 2021). In their review article, Khodadadi et al. discuss the transcription factor NFE2L1/NRF1 as a central regulator of proteasomal function. The authors describe how NFE2L1 controls proteasome biogenesis, interacts with autophagy and mitophagy pathways, and influences processes such as ferroptosis, all within the context of neuronal physiology and neurodegenerative disease.

Neuronal proteostasis also critically depends on the integrity and functionality of specific proteases, including mitochondrial enzymes such as the m-AAA protease (Patron et al., 2018). Loss of its catalytic subunit, AFG3L2, disrupts mitochondrial proteostasis, respiration, and calcium homeostasis, with pathogenic mutations causing spinocerebellar ataxia type 28 (SCA28) and spastic ataxia type 5 (SPAX5) (Di Bella et al., 2010; König et al., 2016; Pierson et al., 2011). In their original research article, Oeztuerk et al. employ a multi-omics-based phenotyping approach to analyze AFG3L2-mutant lymphoblasts derived from a SPAX5 patient. They identify a broad spectrum of mitochondria-associated perturbations, including impaired calcium handling, dysregulation of cytoskeletal organization and vesicle transport, as well as alterations in lipid and steroid metabolism. These findings illustrate the extensive downstream consequences of impaired mitochondrial proteostasis on cellular homeostasis.

A complementary perspective on the interplay between lipid and steroid metabolism and proteostasis is provided by the mini-review article from Pereira Sena et al. While disturbances in lipid metabolism—particularly the disease-modifying role of apolipoprotein E—are well-established in neurodegenerative disorders such as Alzheimer's disease (He et al., 2025; Jackson et al., 2024), the authors broaden this view to encompass both sporadic conditions, including cerebral amyloid angiopathy, and monogenic neurodegenerative disorders such as polyglutamine spinocerebellar ataxias. They discuss how dysregulation of lipid homeostasis can negatively impact proteostasis and thereby promote protein misfolding and aggregation across diverse neurodegenerative disease entities.

Finally, in their opinion article, Sokolov et al. explore unconventional strategies aimed at enhancing proteasomal degradation to counteract protein dyshomeostasis in neurons. The proposed approaches—representing potentially untapped future avenues—range from modulation of transcriptional programs governing proteasomal subunit expression, to recruitment and activation of free 20S proteasomal cores, targeting proteaphagy and proteasomal subunit turnover, and augmenting proteostatic capacity through xenogeneic systems such as disaggregases and archaeal proteasomes. This perspective highlights both the complexity of proteostasis regulation and the breadth of innovative strategies that may be harnessed for therapeutic benefit.

In summary, this Research Topic presents current advances in our understanding of proteostasis disruption in neurodegenerative disorders and highlights emerging concepts that link protein misfolding, impaired degradation, metabolic dysregulation, and neuronal vulnerability. Collectively, the contributions underscore the importance of targeting fundamental proteostatic mechanisms and provide inspiration for future research aimed at developing therapeutic strategies that address one of the central drivers of neurodegeneration: the accumulation of damaging misfolded and aggregated proteins.
